# Elucidation of Chemical Interactions between Crude Drugs Using Quantitative Thin-Layer Chromatography Analysis

**DOI:** 10.3390/molecules27030593

**Published:** 2022-01-18

**Authors:** Naohiro Oshima, Maho Saito, Mina Niino, Yuki Hiraishi, Kana Ueki, Kazuki Okoshi, Takashi Hakamatsuka, Noriyasu Hada

**Affiliations:** 1 Faculty of Pharmaceutical Sciences, Tokyo University of Science, Chiba 278-8510, Japan; 3A17053@ed.tus.ac.jp (M.S.); 3A18059@ed.tus.ac.jp (M.N.); 3B20560@ed.tus.ac.jp (Y.H.); shinkanasen@gmail.com (K.U.); kz_okoshi@rs.tus.ac.jp (K.O.); 2 Division of Pharmacognosy, Phytochemistry and Narcotics, National Institute of Health Science, Kawasaki 210-9501, Japan; thakama@nihs.go.jp

**Keywords:** thin-layer chromatography, herbal-pair theory, chemical interaction, crude drugs

## Abstract

To elucidate the interactions between crude drugs in Kampo medicines (traditional Japanese medicines), it is important to determine the content of the constituents in a cost-effective and simple manner. In this study, we quantified the constituents in crude drug extracts using thin-layer chromatography (TLC), an inexpensive and simple analytical method, to elucidate the chemical interactions between crude drugs. We focused on five crude drugs, for which quantitative high-performance liquid chromatography (HPLC) methods are stipulated in the Japanese Pharmacopoeia XVIII (JP XVIII) and compared the analytical data of HPLC and TLC, confirming that the TLC results corresponded with the HPLC data and satisfied the criteria of JP XVIII. (*Z*)-ligustilide, a major constituent in Japanese Angelica Root, for which a method of quantification has not been stipulated in JP XVIII, was also quantitatively analyzed using HPLC and TLC. Furthermore, Japanese Angelica Root was combined with 26 crude drugs to observe the variation in the (*Z*)-ligustilide content from each combination by TLC. The results revealed that combinations with Phellodendron Bark, Citrus Unshiu Peel, Scutellaria Root, Coptis Rhizome, Gardenia Fruit, and Peony Root increased the (*Z*)-ligustilide content. Quantifying the constituents in crude drug extracts using the inexpensive and simple TLC method can contribute to elucidating interactions between crude drugs in Kampo medicines, as proposed by the herbal-pair theory.

## 1. Introduction

Kampo medicine is a traditional Japanese medicine that consists of an appropriate combination of multiple crude drugs. Its value has been recognized worldwide based on the accumulation of scientific evidence through extensive use. Generally, during the decoction process of Kampo medicines, multiple crude drugs are co-decocted and expected to exert synergistic effects. However, reports on the significance of combining the crude drugs of Kampo medicines, i.e., the herbal-pair theory, are limited. Previously, we have pioneered the elucidation of the interactions among the crude drugs in orengedokuto, a Kampo medicine, and revealed the chemical interactions [1,2] that elicited anti-inflammatory effects [3,4]. These studies indicated the importance of easily determining the content of the constituents in the extract. For continuous research and development regarding the interactions between crude drugs, a simple and inexpensive method of quantification is required because not all research institutions have expensive analytical equipment, such as high-performance liquid chromatography (HPLC) and liquid chromatography/mass spectrometry.

Thin-layer chromatography (TLC) is a well-known and simple analytical method that does not require expensive equipment. Recently, quantitative analysis via TLC has become possible owing to improvements in image recognition capability [5]. Therefore, TLC analysis has attracted attention as a versatile quantitative method. Previously, studies on the quantitative analysis of compounds using TLC have been reported; e.g., berberine from *Coptis teeta* [6] and *Coscinium fenestratum* [7], barakol from *Senna siamea* [8], phyllanthin and gallic acid [9], and saikosaponins [10]. However, information on the content of constituents in hot water extracts and their combination which are frequently used in clinical practice is limited. 

(*Z*)-Ligustilide is a major essential oil bearing phthalide skeleton in Japanese Angelica Root—the root of *Angelica acutiloba* or *A. acutiloba* var. *sugiyamae* (Umbelliferae) stipulated in the Japanese Pharmacopoeia XVIII (JP XVIII) [11]. Many studies on the pharmacological effects of (*Z*)-ligustilide, including antioxidant [12], analgesic [13], platelet aggregation reduction [14], and uterine smooth muscle contraction inhibition [15], have been reported. (*Z*)-Ligustilide is considered the active ingredient in Japanese Angelica Root-containing Kampo medicines such as tokito, tokishakuyakusan, kamishoyosan, juzentaihoto, and kamikihito, which are frequently used in the treatment of gynecological disorders. However, changes in the content of (*Z*)-ligustilide extracted from the combination of crude drugs have not been clarified. 

In this study, we applied TLC analysis to determine the content of constituents in a hot water extract of crude drugs in order to understand the chemical interactions between crude drugs. We first focused on crude drugs, whose constituents were quantified using HPLC methods stipulated in the JP XVIII, and examined them for sufficient analysis using TLC. Next, we analyzed (*Z*)-ligustilide, a major constituent of Japanese Angelica Root, for which no quantitative method has been described in JP XVIII, and compared the TLC data with HPLC data. In addition, we combined Japanese Angelica Root with crude drugs to analyze (*Z*)-ligustilide content using TLC to identify the crude drugs that increase the (*Z*)-ligustilide content.

## 2. Results

### 2.1. Comparison of TLC and HPLC Analytical Data

We analyzed marker compounds for the quality control of five crude drug extracts, which are dried powders obtained from decoctions, namely Ginger, Peach Kernel, Apricot Kernel, Glycyrrhiza, and Phellodendron Bark, using the JP XVIII-stipulated HPLC quantification method. In addition, TLC analysis was performed according to modified HPLC conditions, and the TLC and HPLC analytical data were compared (Table 1). As a result, all crude drugs analyzed using each method satisfied the criteria of the marker compounds, and there was not a significant difference between the TLC and HPLC results. Next, we prepared hot water extracts of the five crude drugs and compared the TLC and HPLC analytical results (Table 2). No remarkable differences were observed in the results obtained by the different analytical methods; particularly for Phellodendron Bark, no significant differences were found. Furthermore, to confirm the reproducibility of the quantitative TLC analysis, the intra- and inter-day precisions were calculated, resulting in values of less than 3.39% and 7.88%, respectively, for all analyses.

### 2.2. Analysis of Japanese Angelica Root-Derived (Z)-Ligustilide 

Focusing on Japanese Angelica Root, the content of (*Z*)-ligustilide was determined and compared using HPLC and TLC. First, we confirmed the precisions of (*Z*)-ligustilide dissolved in methanol by TLC analysis. The intra- and inter-day precisions of (*Z*)-ligustilide itself for TLC analysis were calculated as 10.9% and 15.8%, respectively. Next, HPLC and TLC pretreatments of the dried powder from hot water extract were performed by dissolution in methanol and by partition with ethyl acetate, respectively, and the extraction recovery tests showed 115.9% and 95.8% recovery, respectively. Using the pretreatment method, the contents of (*Z*)-ligustilide in the hot water extract were determined to be 0.00388 ± 0.00009% (HPLC) and 0.00219 ± 0.00026% (TLC), showing no markable difference. The coefficients of variation for HPLC and TLC were less than 2.3% and 12%, respectively. The intra- and inter-day precisions of (*Z*)-ligustilide in the hot water extract for TLC analysis were calculated as 17.0% and 19.8%, respectively. These facts indicated that TLC analysis can be used as a substitute for HPLC analysis of (*Z*)-ligustilide in the extract. 

### 2.3. Combination with Crude Drugs to Increase the Extracted (Z)-Ligustilide Content 

To clarify the chemical interactions between Japanese Angelica Root and other crude drugs, two types of extracts were prepared: a combined formula extract (CF), which is a dried extract of combined crude drugs decocted in hot water, and a blended formula extract (BF) that is a mixture of each dry extract in the corresponding crude drug proportions.

The spot area ratios of (*Z*)-ligustilide in BF and CF were screened by TLC analysis. The results showed that the extraction efficiency of (*Z*)-ligustilide increased by more than 150% when Phellodendron Bark, Citrus Unshiu Peel, Scutellaria Root, Coptis Rhizome, Gardenia Fruit, or Peony Root were combined with Japanese Angelica Root (Figure S1). We analyzed these combinations using the calibration curve and compared the HPLC and TLC results. In the combinations with Coptis Rhizome and Peony Root, the results of HPLC analysis were similar to those of TLC (Table 3). In the combination with Gardenia Fruit, an increased extraction efficiency of (*Z*)-ligustilide was observed via both HPLC and TLC, although HPLC showed a slightly higher value of 241% compared to 193% obtained by TLC. Furthermore, we compared the HPLC and TLC results of each combination that did not show an increase in the extraction efficiency in the screening test, such as Alisma Tuber, Cnidium Rhizome, and Poria Sclerotium. As a result, there was no remarkable difference between the HPLC and TLC data for each combination (Table 3). Meanwhile, the HPLC analyses of Citrus Unshiu Peel, Phellodendron Bark, and Scutellaria Root were difficult because of the overlapping peaks of (*Z*)-ligustilide and each constituent (data not shown). 

## 3. Discussion

We conducted quantitative analysis by TLC, focusing on crude drugs for which quantitative methods are stipulated in the JP XVIII, and compared the results with HPLC data to verify the validity of the TLC data. The values by both methods were generally correspondent and the precision of the TLC analysis was high, indicating that TLC can be substituted for HPLC, which is commonly used to determine the content of the constituents in crude drugs. However, due to the limited number of crude drugs we compared and the statistically significant differences in some of the comparative data, further studies comparing the HPLC and TLC analytical data for other crude drugs are needed to continue to validate quantitative TLC.

(*Z*)-Ligustilide in the hot water extract of Japanese Angelica Root was quantified to be 0.00388% by HPLC and 0.00219% by TLC. Meanwhile, the content percentage of (*Z*)-ligustilide from the methanolic solution of Japanese Angelica Root was approximately 0.05–0.3% [16,17]. These facts indicated that (*Z*)-ligustilide was reduced during the decoction process by hot water. Originally, Japanese Angelica Root was often used in a pulverized powder form of the crude drug itself, not in an extract form, which is a dried powder in hot water extract. Thus, it is desirable to use Japanese Angelica Root in a powder form if the pharmacological effect of Japanese Angelica Root is dependent on (*Z*)-ligustilide. To investigate this, it is necessary to quantify and compare each constituent in powder and extract form. In addition, although there are many reports on the quantification of the constituents in crude drug extracts, most of the previous quantitative analyses have been conducted for organic solvent extracts, especially methanolic extracts, and information on the content of constituents in hot water extract used in clinical practice is limited. Therefore, the quantification of constituents in hot water extracts of crude drugs will also need to be achieved.

The extraction efficiency of (*Z*)-ligustilide from Japanese Angelica Root increased when combined with Phellodendron Bark, Citrus Unshiu Peel, Scutellaria Root, Coptis Rhizome, Gardenia Fruit, or Peony Root. This result suggested that the π–π interaction between (*Z*)-ligustilide and aromatic compounds rich in these crude drugs, increased the solubility of (*Z*)-ligustilide. In fact, it has been reported that the combination of several aromatic curcuminoids increased their solubility and enhanced their nematicidal activities compared to single compounds [18]. Thus, the increased extraction efficiency of (*Z*)-ligustilide by its combination with crude drugs may be attributed to the π–π interactions.

We confirmed that TLC could be used to quantify constituents in crude drug extracts and that it could contribute to the elucidation of chemical interactions between crude drugs. Quantitative TLC studies are easier for many researchers who do not specialize in analytical science to conduct quantitative analysis of crude drugs, with the aim of continuing research and development in the interaction of crude drugs in Kampo medicines, thus contributing to enhancing clarity in the herbal-pair theory.

## 4. Materials and Methods

### 4.1. Experimental Materials

#### 4.1.1. Crude Drugs

Japanese Angelica Root (Lot. 6F08M), Peony Root (Lot. 6F16M), and Rehmannia Root (Lot. EB0025) were purchased from Daiko Shoyaku Co., Ltd. (Nagoya, Japan). Japanese Angelica Root (Lot. G0U0330), Glycyrrhiza (Lot. 9AE0114, I940151), Peach Kernel (Lot. B420320), Apricot Kernel (Lot. EBT0123, I180123), Ginseng (Lot. E1E0403), Atractylodes Lancea Rhizome (Lot. F500239), Cinnamon Bark (Lot. F2T0129), Scutellaria Root (Lot. B420015), Cnidium Rhizome (Lot. F6F0233), Ginger (Lot. F4J0227), Citrus Unshiu Peel (Lot. G830312), Coptis Rhizome (Lot. K7T0018), Gardenia Fruit (Lot. 8151104), Ephedra Herb (Lot. 05B0601), Alisma Tuber (Lot. E8T0304), Asiasarum Root (Lot. F1T0202), Astragalus Root (Lot. A2T0014), Pueraria Root (Lot. F4T0108), Perilla Herb (Lot. 7A09M), Aconite Root (Lot. F990243), Pinellia Tuber (Lot. 6C03M), Moutan Bark (Lot. F450521), Glehnia Root and Rhizome (Lot. H5F0518), Poria Sclerotium (Lot. 122404), and Phellodendron Bark (Lot. ABD0016) were purchased from Uchida Wakanyaku Ltd. (Tokyo, Japan). 

#### 4.1.2. Materials

The following compounds were purchased from FUJIFILM Wako Pure Chemical Co. (Osaka, Japan): [6]-gingerol (Lot. APQ6161), amygdalin (Lot. KPM0958), glycyrrhizin (Lot. CAH4202), berberine (Lot. PTM0089), and (*Z*)-ligustilide (Lot. ESJ5922). The solvents used for HPLC and TLC analyses were of research grade. The TLC plate (Silica Gel 60 F254) was purchased from Merck Co. (Darmstadt, Germany).

#### 4.1.3. Equipment

The HPLC equipment (Shimadzu Co., Kyoto, Japan) comprised the following components: Degasser, DGU-20A_3R_, Pump; LC-20AD, Autosampler; SIL-20A, column oven; CTO-20A, UV-VIS Detector; SPD-20A. 

The Kubota 5200 (KUBOTA Co., Tokyo, Japan) centrifuge was used. 

#### 4.1.4. Preparation of Hot Water Extracts

A total of 20 g of a cut single crude drug or a combination of two mixed crude drugs with a crude drug ratio of 1:1 (combined formula extract: CF) was weighed, placed in a decoction pot (HARIO Co., Ltd., Tokyo, Japan), and decocted with 400 mL of purified water until the volume was reduced to approximately half of the original volume. The extract was filtered and centrifuged at 3000 rpm for 10 min, and the supernatant was freeze-dried to obtain a dried extract. 

The dried extracts were blended in a 1:1 ratio of crude drugs; the extract weight per gram of crude drug was determined, and the mixture was used as a blended formula extract (BF). 

### 4.2. Quantitative Analysis

#### 4.2.1. Marker Compounds for Quality Control Stipulated by JP XVIII

##### HPLC

We used this method of quantification according to JP XVIII (Table 4 and Figure S2).

##### TLC

**Figure 1:**
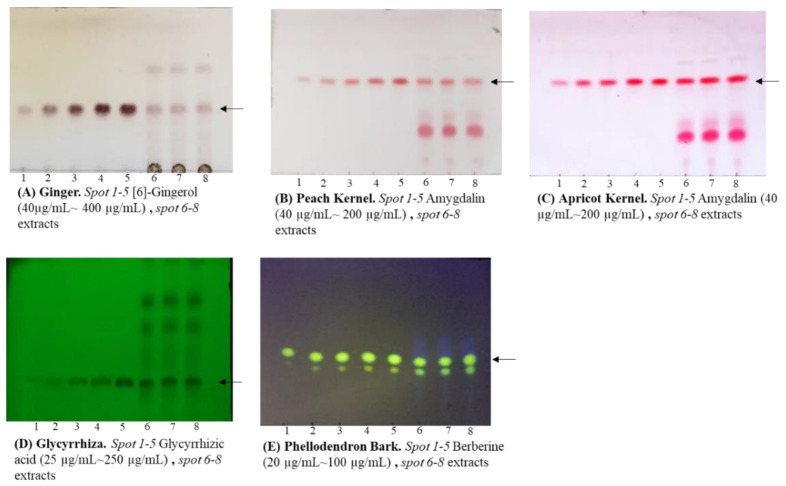
TLC images obtained using modified methods according to the identification test in JP XVIII.

We used a modified method of analysis according to the identification test described in JP XVIII (Figure 1, Table 5) and the TLC images were analyzed using the software JUST TLC^®^ (Sweday Co., Södra Sandby, Sweden).

#### 4.2.2. (*Z*)-Ligustilide

##### HPLC

- Pretreatment for Analysis of Hot Water Extracts

In a single hot water extract, blended formula extract, and combined formula extract, a content of each dried extract equivalent to 200 mg of Japanese Angelica Root extract was weighed, methanol was added to adjust the volume to 20 mL using a volumetric flask, and then the solution was filtered. The filtrate was used as the analytical sample.

- HPLC Analysis

The analytical conditions for (*Z*)-ligustilide were as follows: column, Intersil ODS-3 (*φ* 4.6 × 150 mm, 5 µm), solvent; (A) H_2_O; (B) 0.1% HCOOH in CH_3_CN; flow rate, 1 mL/min; gradient mode, B 50% → 100% (30 min) → 100% (45 min) → 50% (45.01 min) → 50% (60 min). The oven temperature was 40 °C, injection volume was 10 µL, and wavelength was 326 nm. For detailed information, see Figure S3.

##### TLC

- Pretreatment for the Analysis of Hot Water Extracts

The hot water extract (100 mg) was shaken with 100 mL of water; thereafter, 100 mL of ethyl acetate was added. The solution was then shaken. The ethyl acetate layer was evaporated under reduced pressure, the residue was dissolved in methanol (0.5 mL), and the solution obtained was used as the sample solution.

- Analysis

**Figure 2:**
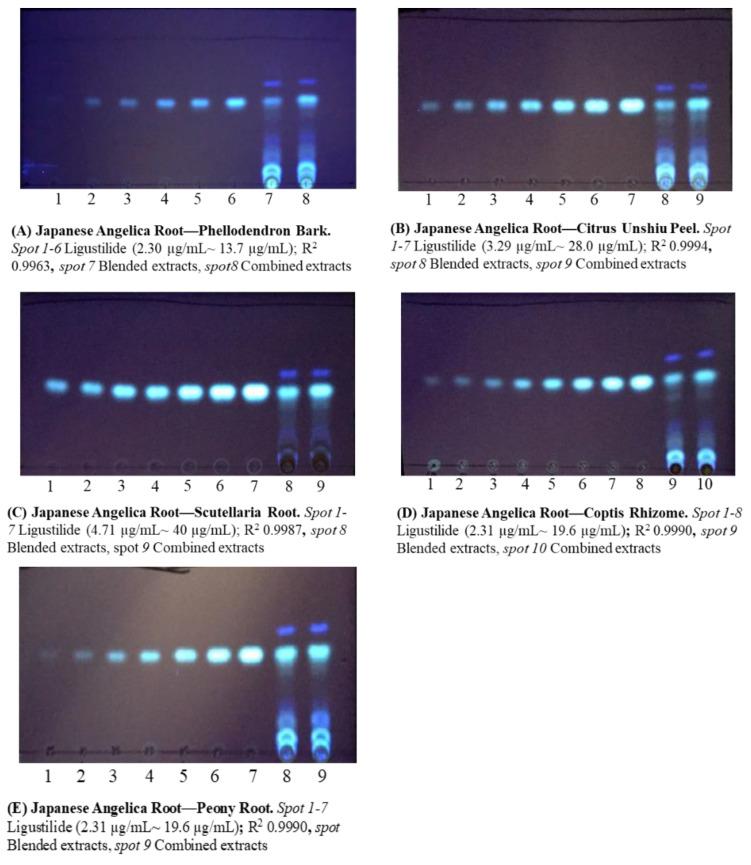
TLC images of (*Z*)-ligustilide in combinations of Japanese Angelica Root and crude drugs.

Twenty microliters of the methanol solution of (*Z*)-ligustilide for the standard curve, BF, and CF were applied to a TLC plate, and the plate was developed with *n*-hexane/ethyl acetate solution (10:3, *v*/*v*). After development, the dried plate was irradiated with UV light (*λ* 365 nm) (Figure 2).

A concentration range was set for each analysis, and the spot area of the target compounds was confirmed to be within the range of the calibration curve. The rate of change in the concentration of extracted (*Z*)-ligustilide was calculated as the concentration ratio of CF/BF.

### 4.3. Statistics

Results are expressed as the mean ± standard deviation (S.D.) The statistical significance of difference between any two groups was calculated by paired Student’s *t*-test. The criterion of significance was set as *p* < 0.001.

## 5. Conclusions

This study shows that constituents in two extracts that were prepared according to the modified JP XVIII and decocted in hot water can be quantitatively analyzed by TLC, based on the comparison of HPLC analytical data of five crude drugs and the quantitative TLC analysis of (*Z*)-ligustilide in hot water extract of Japanese Angelica Root, for which quantitative methods are not stipulated in the JP XVIII. Moreover, changes in the extraction efficiency of (*Z*)-ligustilide depending on the combination of Japanese Angelica Root with 26 crude drugs were analyzed by TLC and HPLC to identify the crude drugs that increased the extraction efficiency by more than 150%. As a result, TLC analysis could satisfy the criteria of the marker compounds and precisely analyze the constituents. No markable differences were observed between the TLC and HPLC analytical data, and good precision results by TLC were obtained. In addition, (*Z*)-ligustilide content in hot water extract of Japanese Angelica Root was revealed to be 0.00219%, indicating that TLC analysis could quantify extremely small amounts with inter-day precisions of less than 19.8%. Furthermore, each of the 26 crude drugs could be combined with Japanese Angelica Root. In particular, Phellodendron Bark, Citrus Unshiu Peel, Scutellaria Root, Coptis Rhizome, Gardenia Fruit, and Peony Root increased the content of (*Z*)-ligustilide. Additionally, we found that TLC analysis could quantify even if peaks overlap in HPLC. As suggested by the herbal-pair theory, the determination of constituents in crude drug extracts by TLC which is an inexpensive and simple method, contributes to the elucidation of the chemical interactions between crude drugs consisting of Kampo formulae.

## Figures and Tables

**Table 1:** Comparison of the contents of marker compounds stipulated by Japanese Pharmacopoeia.

Crude Drugs	Marker Compounds	Content by HPLC (%) ^#^	Content by TLC (%) ^#^	Criteria for JP18	*t*-Values
Ginger	[6]-Gingerol	0.41 ± 0.00%	0.94 ± 0.06%	≧0.3%	25.6
Peach Kernel	Amygdalin	1.94 ± 0.01%	2.47 ± 0.15%	≧1.2%	4.83
Apricot Kernel	Amygdalin	4.82 ± 0.03%	4.15 ± 0.15%	≧2.0%	5.25
Glycyrrhiza	Glycyrrhizin	4.15 ± 0.01%	4.44 ± 0.15%	≧2.0%	3.28
Phellodendron Bark	Berberine	3.99 ± 0.01%	2.92 ± 0.23% *	≧1.2%	6.43

* TLC images were analyzed by negative mode; ^#^ Mean ± standard deviation (*n* = 3); The HPLC and TLC data of the crude drugs were not significantly different using a standard *t*-test, assuming a normal distribution at α = 0.001.

**Table 2:** Content and precisions of the marker compounds in hot water extracts.

Crude Drugs	Marker Compounds	Content by HPLC (%) ^#^	Content by TLC (%) ^#^	Intra-Day Precisions	Inter-Day Precisions	*t*-Values
Ginger	[6]-Gingerol	0.68 ± 0.06%	0.92 ± 0.10%	1.50%	7.88%	6.42
Peach Kernel	Amygdalin	25.6 ± 0.17%	22.9 ± 1.19%	0.49%	2.20%	11.9
Apricot Kernel	Amygdalin	29.1 ± 0.12%	34.0 ± 0.43%	1.01%	2.07%	23.3
Glycyrrhiza	Glycyrrhizin	6.3 ± 0.14%	8.88 ± 0.11%	3.39%	3.13%	22.5
Phellodendron Bark	Berberine	10.8 ± 0.32% *	10.25 ± 0.55% *	1.28%	6.10%	3.01

^#^ Mean ± standard deviation (*n* = 3); * Not significantly different using a standard *t*-test, assuming a normal distribution at α = 0.001.

**Table 3:** Combination of Japanese Angelica Root with a crude drug.

Combinations between Japanese Angelica Root and a Crude Drug	Ligustilide Ratio of CF/BF by HPLC (%)	Ligustilide Ratio of CF/BF by TLC (%)
+Phellodendron Bark	—	175%
+Citrus Unshiu Peel	—	200%
+Scutellaria Root	—	234%
+Coptis Rhizome	176%	172%
+Gardenia Fruit	241%	193%
+Peony Root	141%	153%
+Alisma Tuber	128%	112%
+Cnidium Rhizome	105%	108%
+Poria Sclerotium	72.0%	77.0%

—: Overlapping.

**Table 4:** HPLC analysis conditions of marker compounds stipulated by JP18.

Crude Drugs	Target Compounds	Retention Time	Column	Solvent (Isocratic Mode)	Wavelength	Oven Temperature
Ginger	[6]-Gingerol	19 min	Inertsil ODS-3 (*φ* 4.6 × 150 mm, 5 µm, GL Sciences Inc.)	Water:MeCN:Phosphoric acid = 3800:2200:1	205 nm	40 °C
Peach Kernel	Amygdalin	12 min		Sodium dihydrogen phosphate (0.05 mol/L):MeOH = 5:1	210 nm	45 °C
Apricot Kernel	Amygdalin	12 min		Sodium dihydrogen phosphate (0.05 mol/L):MeOH = 5:1	210 nm	45 °C
Glycyrrhiza	Glycyrrhizin	15 min		Water:MeCN:Acetic acid = 720:280:5	254 nm	40 °C
Phellodendron Bark	Berberine	10 min		Sodium dihydrogen phosphate (3.4 g) and Sodium dodecyl sulfate (1.7 g) dissolved in a mixture of water-MeCN (1/1, *v*/*v*, 1000 mL)	345 nm	40 °C

**Table 5:** TLC analysis conditions of the marker compounds.

Crude Drugs	Target Compounds	Extraction Method	Developing Solvent	Detection
Ginger	[6]-Gingerol	To a Ginger powder (1 g), 30 mL of a methanol/water mixture (3:1) was added, shaken for 20 min, centrifuged, and the supernatant was separated. To the residue, 30 mL of a methanol/water mixture (3:1) was added, and these procedures were repeated two more times. All extracts were combined and methanol/water mixture (3:1) was added to make an accurate volume of 100 mL.	Ethyl acetate/*n*-hexane (1/1, *v*/*v*)	Sprayed with 4-dimethylaminobenzaldehyde, followed by heating at 105 °C
Peach Kernel	Amygdalin	To 0.5 g of crude drug powder, add 40 mL of diluted methanol (9 → 10), heat for 30 min on a water bath equipped with a reflux cooler. After filtration, it was added diluted methanol (9 → 10) to adjust exactly 50 mL. To 5 mL of this solution, water was added to make 10 mL, and the mixture was filtered.	Ethyl acetate/MeOH/Water (20/5/4, *v*/*v*/*v*)	Sprayed with thymol-sulfuric acid methanol solution, followed by heating at 105 °C
Apricot Kernel	Amygdalin			
Glycyrrhiza	Glycyrrhizin	Add 70 mL of diluted ethanol to 0.5 g of Glycyrrhiza powder, shake for 15 min, centrifuge, and separate the supernatant liquid. To the residue, 25 mL of diluted ethanol was added, and the same operation was performed. All the extracts were combined and diluted ethanol was added to adjust 100 mL.	*n*-Butanol/Water/Acetic acid (7/2/1, *v*/*v*/*v*)	Irradiated with UV light (*λ* 254 nm)
Phellodendron Bark	Berberine	A total of 0.5 g of Phellodendron Bark powder was weighed, 30 mL of methanol/diluted hydrochloric acid mixture (100:1) was added, and the mixture was heated in a water bath with a reflux cooler for 30 min. The residue was repeated twice with 30 mL and 20 mL of a methanol/diluted hydrochloric acid mixture (100:1). To the last residue, 10 mL of methanol was added, shaken sufficiently, and filtered. All filtrates were combined and methanol was added to adjust to an accurate volume of 100 mL.		Irradiated with UV light (*λ* 365 nm)

TLC; normal phase; developing distance 5 cm.

## Data Availability

Data is contained within the article and Supplementary Materials.

## Supplementary Materials

The following are available online. Figure S1: Screening for the extraction efficiency of (Z)-ligustilide in combinations of Japanese Angelica Root with several crude drugs; Figure S2: HPLC chromatograms obtained using JP XVIII-stipulated quantitative methods; Figure S3: HPLC chromatograms of (*Z*)-ligustilide in combinations of Japanese Angelica Root with crude drugs.

## Sample Availability

Samples of the compounds are available from the authors.
