# Supplementary material for: Elucidation of Chemical Interactions between Crude Drugs Using Quantitative Thin-Layer Chromatography Analysis
Source: Molecules. 2022 Jan 18;27(3):593. doi: 10.3390/molecules27030593 (PMC8839786; doi:10.3390/molecules27030593)
Supplement: Supplementary file 1 [file molecules-27-00593-s001.zip › molecules-1514237-Supplementary.pdf]

Supplementary Material

Elucidation of Chemical Interactions between Crude Drugs using Quantitative Thin-Layer Chromatography Analysis

Naohiro Oshima [1,*], Maho Saito [1], Mina Niino [1], Yuki Hiraishi [1], Kana Ueki [1], Kazuki Okoshi [1], Takashi Hakamatsuka [2] and Noriyasu Hada [1,*]

[1] Faculty of Pharmaceutical Sciences, Tokyo University of Science, Chiba 278-8510, Japan; 3A17053@ed.tus.ac.jp (M.S); 3B20560@ed.tus.ac.jp (Y. H.); 3A18059@ed.tus.ac.jp (M. N.); kz_okoshi@rs.tus.ac.jp (K. O.).

[2] Division of Pharmacognosy, Phytochemistry and Narcotics, National Institute of Health Science, Kawasaki, 210-9501, Japan; thakama@nihs.go.jp (T. H.).

* Correspondence: hada@rs.tus.ac.jp; Tel.: +81-47-121-3612 (N. H.), n-oshima@nihs.go.jp (N. O.)

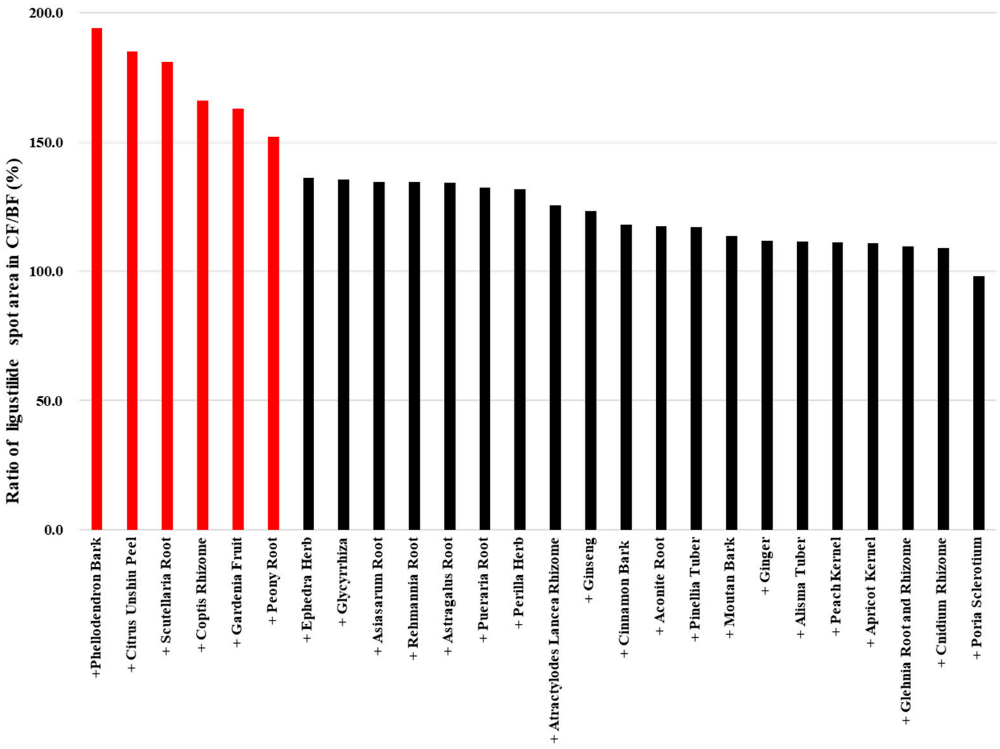

Figure S1. Screening for the extraction efficiency of (*Z*)-ligustilide in combinations of Japanese Angelica Root with several crude drugs. n=1.

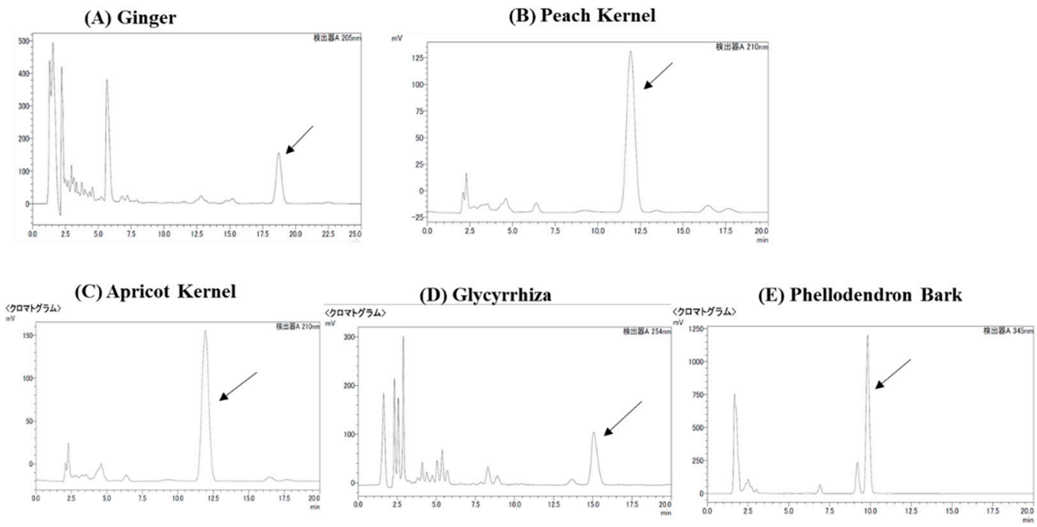

Figure S2. HPLC chromatograms obtained using JP XVIII-stipulated quantitative methods

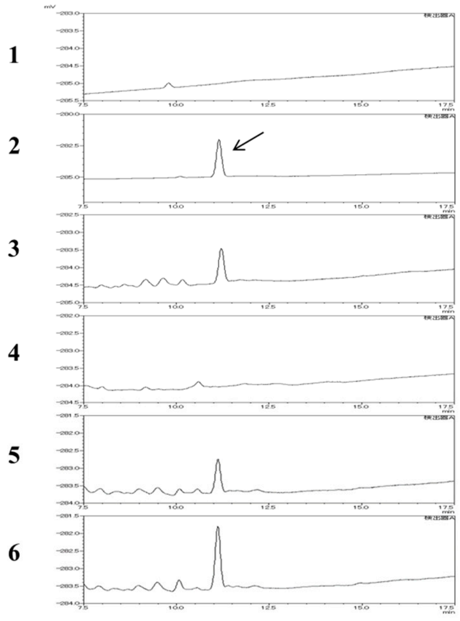

(A) Japanese Angelica Root—Coptis Rhizome. *1* Blank, *2* Ligustilide, *3* Japanese Angelica Root extract, *4* Coptis Rhizome extract, *5* Blended formula, *6* Combined formula. $R^2 = 0.9999$ (0.3–1.6 μg/mL),

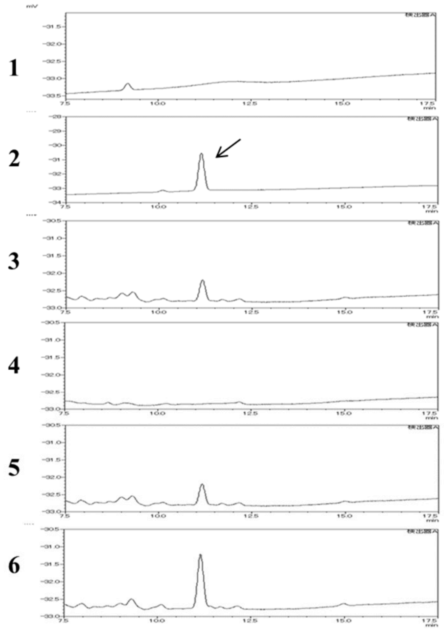

(B) Japanese Angelica Root—Gardenia Fruit. *1* Blank, *2* Ligustilide, *3* Japanese Angelica Root extract, *4* Gardenia Fruit extract, *5* Blended formula, *6* Combined formula. $R^2 = 0.9999$ (0.3–1.6 μg/mL),

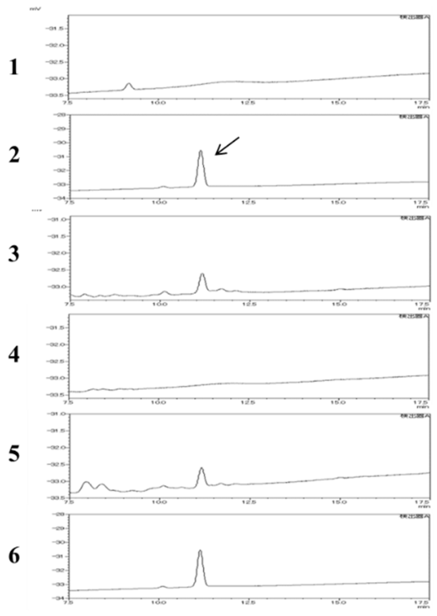

(C) Japanese Angelica Root—Peony Root. *1* Blank, *2* Ligustilide, *3* Japanese Angelica Root extract, *4* Peony Root extract, *5* Blended formula, *6* Combined formula. $R^2 = 0.9999$ (0.3–1.6 μg/mL),

Figure S3. HPLC chromatograms of (*Z*)-ligustilide in combinations of Japanese Angelica Root with crude drugs
